# Clinicopathological characteristics and predictors of outcome of rapidly progressive glomerulonephritis: a retrospective study

**DOI:** 10.1186/s12882-024-03532-y

**Published:** 2024-03-18

**Authors:** Osama Nady Mohamed, Sharehan Abdelrahman Ibrahim, Rabeh Khairy Saleh, Ahmed S. Issa, Amr Setouhi, Ayman Ahmed Abd Rabou, Mahmoud Ragab Mohamed, Shaimaa F. Kamel

**Affiliations:** 1 Department of Internal Medicine, Faculty of medicine, Minia University, Minia, Egypt; 2 Department of Pathology, Faculty of medicine, Minia University, Minia, Egypt; 3 Department of Radiology, Faculty of medicine, Minia University, Minia, Egypt; 4 Department of Cardiology, Faculty of medicine, Minia University, Minia, Egypt; 5 Department of Clinical Pathology, Faculty of medicine, Minia University, Minia, Egypt

**Keywords:** End stage renal disease, Glomerulonephritis, Hemodialysis, Interstitial fibrosis with tubular atrophy

## Abstract

**Background:**

Globally, there are regional and time-based variations in the prevalence, etiology, and prognosis of rapidly progressive glomerulonephritis (RPGN). Prognosis of RPGN is poor, with a higher risk of death and end stage renal disease (ESRD) even with immunosuppressive medications. In the Middle East and North Africa, the studies on this disease are very limited. Therefore, we determined the predictors of outcome of RPGN.

**Methods:**

We retrospectively assessed 101 adult patients over age of 18, diagnosed with RPGN based on renal biopsy illustrating crescents in ≥ 50% of the glomeruli. Patients who had crescents in their renal biopsies that were < 50% and those who refused to consent to a renal biopsy were excluded. We categorized the patients into 3 groups based on immunohistochemistry; type I, type II and type III. Then, depending on renal loss, we divided them into ESRD and non-ESRD groups. The clinical history and physical examination were retrieved. Additionally, 24-hour urine protein, urine analysis, renal function tests, serum albumin, complete blood count, antinuclear antibodies, anti-double stranded DNA antibodies, ANCA antibodies and serum complement levels were checked. Each patient underwent a kidney biopsy for immunohistochemistry and light microscopy. The percentage of crescentic glomeruli, number of sclerosed glomeruli, tertiary lymphoid organ (TLO), neutrophil infiltration, endocapillary or mesangial hypercellularity, interstitial fibrosis with tubular atrophy (IFTA) were analyzed. Primary outcomes (remission, ESRD and mortality) and secondary outcomes were assessed.

**Results:**

Type II was the most frequent cause of RPGN (47.5%), followed by type III (32.7%) and type I (19.8%). 32 patients (31.7%) died during follow up, whereas 60 patients (59.4%) developed ESRD. In 41 patients (40.6%), remission occurred. Oliguria, serum creatinine, and need for HD at presentation were significantly increased in ESRD group compared to non-ESRD group (*P* < 0.001 for each). Mesangial proliferation, IFTA, TLO formation, sclerotic glomeruli and fibrous crescents were also significantly increased in ESRD group in comparison to non-ESRD group (*P* < 0.001 for each). Glomerulosclerosis (*P* = 0.036), and IFTA (*P* = 0.008) were predictors of ESRD. Infections (*P* = 0.02), respiratory failure (*P* < 0.001), and heart failure (*P* = 0.004) were mortality risk factors.

**Conclusion:**

Type II RPGN was the most common. Infection was the most frequent secondary outcome. Oliguria, glomerulosclerosis, the requirement for hemodialysis at presentation, IFTA and TLO formation were predictors of ESRD. Respiratory failure, heart failure and infections were significant predictors of mortality.

## Introduction

The clinical syndrome known as rapidly progressive glomerulonephritis (RPGN) is characterized by a rapid decline in renal function, frequently accompanied by anuria or oliguria, and glomerulonephritis-specific features including glomerular proteinuria and dysmorphic hematuria [1]. Extensive crescent formation is typical of aggressive GN that results in RPGN. Tإhe pathological term, crescentic glomerulonephritis (GN) and the clinical term RPGN are occasionally used synonymously. A abrupt and progressive loss in renal function and crescents in 50% or more of the glomeruli are histological characteristics of crescentic GN [2]. It is considered a “medical emergency” because end-stage renal failure can develop within a few weeks or months due to the unrelenting reduction in renal function [3]. Some of the clinical manifestations include edema, hematuria, oliguria, and proteinuria that appear suddenly. RPGN is diagnosed based on laboratory findings and a kidney biopsy that reveals the unique crescentic development in Bowman’s capsule. At the time of diagnosis, an increase in serum creatinine has typically also been seen in the majority of cases [4].

Histopathologically, there are four subtypes of RPGN: immune complex, pauci-immune, anti-glomerular basement membrane (Anti-GBM) antibody disease, and idiopathic. Anti-GBM disease (type I) represents 10% of RPGN cases. When there are anti-GBM antibodies present, combinations of alveolar haemorrhage and glomerulonephritis is referred to as the “Goodpasture syndrome.” Anti-GBM disease is the medical term for glomerulonephritis without alveolar haemorrhage that is caused by anti-GBM antibodies. Linear IgG deposits can be seen in renal biopsy after immunohistochemistry (IHC) labelling. Immune complex RPGN (type II) which represents 40% of cases occurs in numerous systemic rheumatic diseases such as lupus nephritis and cryoglobulinemic GN. It is also seen in infectious diseases as post-streptococcal GN, infective endocarditis, visceral abscess and also occurs with other primary glomerular disorders including membranoproliferative glomerulonephritis (MPGN), and IgA nephropathy. IHC staining illustrates granular immunological deposits. Pauci-immune RPGN (type III) which constitutes 50% of cases is characterised by absence of complement or immune complex deposition on IHC staining. The majority of patients also exhibit systemic vasculitis and high antineutrophil cytoplasmic antibodies (ANCAs), typically myeloperoxidase-ANCA or antiproteinase 3-ANCA. Idiopathic RPGN (type IV) is rare. This type includes patients with either of the following: (a) Immune complexes without a clear cause such as an infection, a rheumatic systemic disease, or a glomerular disease. (b) Pauci-immune features without ANCA antibodies [5].

There are regional and temporal variations in the prevalence, etiology, and prognosis of RPGN worldwide [6–9]. RPGN is seen in 4–10% of native kidney biopsies [7]. Despite immunosuppressive medications, its prognosis is poor, with a higher risk of ESRD and death [6]. The prognosis is significantly influenced by specific clinicopathological characteristics, such as the subtype, serum creatinine, oliguria,, age, and an increased percentage of crescentic glomeruli [10]. In the Middle East and North Africa, the studies on this disease are very limited [11]. The disease spectrum of RPGN has been described in only a few previous studies [7], and detailed data from North Africa especially Egypt are unavailable to date. Thus, this study was the first to aim at determining the clinical, histopathological and treatment predictors of outcome and identifying factors of poor prognosis. Early prediction of these risk factors helps in early diagnosis and treatment in order to improve the renal and patient survival.

## Methods

### Study design and population

This retrospective study was carried out in a tertiary University center that included three major hospitals, Internal medicine, Nephrology and Rheumatology hospitals, in Minia, Egypt. We had collected our patients from these major hospitals. The hospital records were examined from January 2015 to July 2022 to detect the patients with crescentic GN and followed for at least 1 year. The study was in accordance with the ethical standards of Institutional Review Board (IRB), Faculty of Medicine, Minia University. Approval No. was 842-7-2023. Informed consent was obtained from all participants of the study. Following institutional ethical committee permission, our hospital performed 3340 renal biopsies, of which 101 (3%) were found to have RPGN. RPGN was diagnosed based on the renal histopathology illustrating crescents in ≥ 50% of the glomeruli. Inclusion criteria for this study included adult patients over age of 18 with RPGN that was confirmed by biopsy and rapid progressing renal failure. Patients who had crescents in their renal biopsies that were < 50% and those who refused to consent to a renal biopsy were excluded.

### Clinical and laboratory data

We retrieved and analyzed the whole detailed clinical history with regard to the symptoms of different organs and complete systemic and physical examination data. We also checked and reviewed the results of the laboratory tests, such as the 24-hour urine protein or spot protein to creatinine ratio, urine microscopy, dipstick analysis, random blood sugar, C-reactive protein, liver function tests, renal function tests, and CBC. The equation developed by the Chronic Kidney Disease Epidemiology Collaboration was used to get the estimated glomerular filtration rate (eGFR). ANCA antibodies have been measured using the Dot-blot strip test and indirect immunofluorescence (IF) test/ELISA, antinuclear antibodies (ANA) have been measured using the indirect IF technique, anti-double-stranded DNA antibodies have been measured using the indirect IF test, and serum complement levels have been measured using nephelometry. All patients had a chest X-ray, an electrocardiogram, and an abdominal ultrasound. Every patient suspected of having heart failure underwent comprehensive two- and three-dimensional transthoracic echocardiography in order to measure the left ventricle’s dimensions and volumes during both diastole and systole, assess the diastolic function based on the Doppler of diastolic mitral flow and tissue Doppler, and accurately measure the left ventricular ejection fraction by Simpson biplane method and M-mode [12]. Each patient underwent an automated biopsy gun-assisted percutaneous kidney biopsy under ultrasound guidance. The sample was prepared for IHC and light microscopy (LM) studies. For LM, renal biopsy samples were preserved in 10% neutral buffered formalin. Histological staining was done on consecutive serial 3 μm samples. The stains used were Masson’s trichrome, Jones silver methenamine, periodic acid-Schiff, and hematoxylin-eosin. The number of glomeruli in total, the percentage of glomeruli with crescents, the number of glomeruli that were entirely sclerosed, neutrophil infiltration, endocapillary or mesangial hypercellularity, the characteristics of vasculitis, the degree of interstitial fibrosis, the amount of tubular atrophy, and fibrinoid necrosis, were all carefully analysed in the renal biopsy. A semiquantitative scoring system based on the degree of vascular blockage in the most seriously affected vessel was used to assess renal arteriosclerosis [13]. Scores of 0, 1, 2, and 3 indicated arterial luminal constriction of < 10%, 10– 25%, 26 − 50%, and > 50%, respectively. TLO development was outlined as a lymphocyte-based organised cluster with an arbitrary cut-off of 50 cells [14]. Severe tubular atrophy was defined as the presence of tubular atrophy in more than 50% of the cortical tubules in a specimen [13]. Thick sections (5 μm) were prepared from the renal biopsy and IHC was performed. Tissue sections were processed according to the routine IHC protocol [15]. Slides were stained by immunoglobulin A (IgA) antibody (polyclonal rabbit antibody, clone 267 A-16, 1mL concentrate, incubated in a humidity chamber for 45 min at 4 ºC (diluted at 1/200), Cell Marque, USA), immunoglobulin G (IgG) antibody (polyclonal rabbit antibody, clone 269 A-16, 1mL concentrate, incubated in a humidity chamber for 45 min at 4 ºC (diluted at 1/200), Cell Marque, USA), and complement 3 (C3) antibody (polyclonal rabbit antibody, clone, HPA020432, 100 µl concentrate, incubated in a humidity chamber for 45 min at 4 ºC (diluted at 1/300), Sigma Aldrich, USA). Staining of IgG, IgA and C3 were detected along glomerular basement membrane and mesangium [16, 17]. The patients were classified based on IHC into type I RPGN, type II RPGN and type III RPGN. We further divided the patients according to the renal loss into ESRD group and non-ESRD group.

### Outcomes

Primary and secondary outcomes of the therapeutic response have been studied. Remission and death were the primary outcomes. A 24 h urine protein less than 0.5 g/day and a serum creatinine less than 1.4 mg/dL were used to define complete remission. Serum creatinine levels that were stable or decreasing in non-dialysis patients, dialysis independence, and serum creatinine levels below 5.8 mg/dL in dialysis dependents were all considered signs of partial remission. In non-dialysis patients, no response was known as an increase in serum creatinine, dialysis dependence, or ESRD and in dialysis dependent patients, as an increase in serum creatinine level more than 5.8 mg/dL or dialysis dependence. Another important primary outcome of the trial was a patient’s death while the patients were being followed up. Endocrinological issues as diabetes mellitus (DM), hematological disorders (neutropenia, anemia or thrombocytopenia), infections, gastrointestinal issues (diarrhoea, vomiting), cardiovascular diseases (heart failure), and dermatological problems (rash, alopecia) were secondary outcomes [2]. The European Society of Cardiology defines heart failure as a clinical syndrome that includes pulmonary crackles, elevated jugular venous pressure, tachycardia, and lower limb edema, along with symptoms like shortness of breath, persistent coughing or wheezing, and swelling in the ankles.

### Treatment

Clinicians created treatment regimens based on their knowledge, the patient’s condition, and previously documented treatment protocols. In general, intravenous cyclophosphamide was administered in conjunction with high dose intravenous methylprednisolone (1000 mg/day) for 3 days to induce remission. Patients were given oral prednisolone 1 mg/kg/day (with a maximum daily dose of 80 mg) following high-dose corticosteroid therapy, with tapering within 6 months. Maintenance therapy consisted of oral azathioprine and low-dose corticosteroids. Patients with further life-threatening involvement, such as diffuse alveolar hemorrhage or serum creatinine levels above 5.7 mg/dL as a result of RPGN, also underwent plasma exchanges as an induction therapy. Rituximab was also used for induction and maintenance therapy [18].

### Statistical analysis

Using SPSS version 25, data analysis was carried out. When displaying continuous data, medians with interquartile ranges (IQR) or mean with standard deviation were used, whereas a number and a percentage were used to construct categorical data. Mann-Whitney test and Kruskal Wallis test were used to evaluate non-parametric continuous variables for comparisons involving two groups or more, respectively. Using the chi-square test, categorical data were compared. To determine the relationship between categorical and continuous variables or two categorical variables, Spearmen correlation was used. The predictors of renal loss and death in the patients were found using logistic regression and Cox regression analyses, respectively. Kaplan-Meier analysis was used to compare the renal and patient survival by treatment regimens.

## Results

### Comparison of demographic and clinical parameters between RPGN groups

From January 2015 to July 2022, our hospital performed 3340 renal biopsies, of which 101 (3%) were discovered to have RPGN. Type II was the most frequent cause of RPGN (47.5%), followed by type III (32.7%) and type I (19.8%). Type II included 34 lupus nephritis patients (33.7%), 6 patients with cryoglobulinemic GN (5.9%), 4 patients with IgA nephropathy (4%), 2 patients with post-infectious GN (2%) and 2 patients with MPGN (2%). Patients with type I, type II, and type III had median ages of 34, 29, and 60 years, respectively. Type III had a significantly higher age compared to type I and type II (*P* < 0.001 for each) (Table 1).

**Table 1:** Demographic and clinical characteristics of the studied RPGN patients

Variables	Type I RPGN *n* = 20	Type II RPGN *n* = 48	Type III RPGN *n* = 33	I vs. II	I vs. III	II vs. III
				*P* value		
Age (years)	34 (13)	29 (10.75)	60 (10)			
	25–44	20–55	45–68	0.54	**< 0.001**	**< 0.001**
Gender (%)						
Male Female	6 (30%) 14 (70%)	14 (29.2%) 34 (70.8%)	23 (69.7%) 10 (30.3%)	0.92	**< 0.001**	**< 0.001**
BMI (Kg/m^2^)	26.5 (1.95) 24.3–29.4	26.4 (2.1) 22.65–29.7	25.8 (3.3) 22–31.64	0.7	0.25	0.37
HTN (%)	14 (70%)	40 (83.3%)	15 (45.5%)	0.21	0.08	**< 0.001**
Edema (%)	6 (30%)	30 (62.5%)	19 (57.6%)	**0.01**	0.051	0.65
Microscopic hematuria (%)	10 (50%)	40 (83.3%)	28 (84.8%)	**0.004**	**0.006**	0.85
Macroscopic hematuria (%)	10 (50%)	8 (16.7%)	5 (15.2%)	**0.004**	**0.006**	0.85
Oliguria (%)	12 (60%)	30 (62.5%)	13 (39.4%)	0.84	0.14	**0.04**
Fever (%)	2 (10%)	18 (37.5%)	2 (6.1%)	**0.02**	0.59	**< 0.001**
Skin rash (%)	2 (10%)	30 (62.5%)	2 (6.1%)	**< 0.001**	0.6	**< 0.001**
Hemoptysis (%)	14 (70%)	8 (16.7%)	8 (24.2%)	**< 0.001**	**0.001**	0.22
Arthritis (%)	4 (20%)	30 (62.5%)	12 (36.4%)	**0.001**	0.2	**0.02**
Need for HD at presentation (%)	14 (70%)	30 (62.5%)	11 (33.3%)	0.4	**< 0.001**	**< 0.001**
Serum creatinine at presentation (mg/dL)	5.25 (4)	4.75 (3.65)	2.7 (2.6)			
	2.3–7.2	1.8–8.0	1.2–5.8	0.43	**0.003**	**0.048**
GFR at presentation (ml/min/1.73^2^)	10.5 (10)	12.5 (18.75)	20 (14)			
	7–28	6–45	9–81	0.45	**0.003**	0.052
Hb (gm/dL)	8.3 (1.6)	8.85 (1.35)	8.9 (2)			
	7.6–10.5	6.9–10	6.6–10.2	0.45	0.97	0.6
Serum albumin (gm/dL)	3 (0.2)	2.1 (1.0)	1.9 (0.4)			
	2.7–3.1	1.6–3.1	1.8–2.6	**< 0.001**	**< 0.001**	**0.04**
Proteinuria (gm/day)	2.8 (0.8)	5.1 (2.1)	4.8 (1.15)			
	2–3.4	3.5–12	3.5–6.8	**< 0.001**	**< 0.001**	0.54

BMI: body mass index, eGFR: estimated glomerular filtration rate, Hb: hemoglobin, HD: hemodialysis, RPGN: rapidly progressive glomerulonephritis

Data were expressed as number (%),or median (Interquartile range) and minimum-maximum

**Fig. 1:**
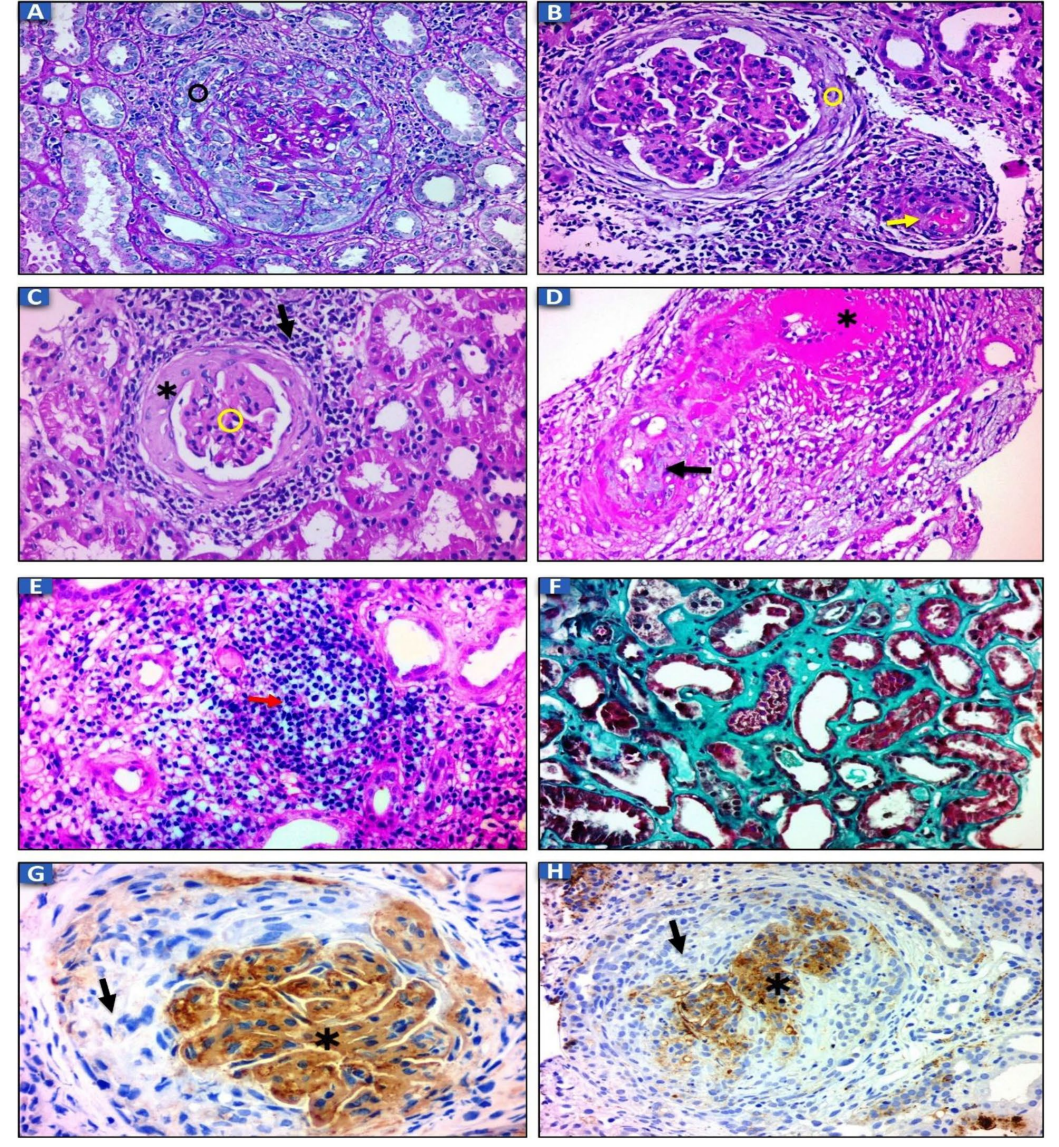
Light microscopic and immunohistochemical changes in patients with RPGN: (**A**) the glomerulus showed cellular crescent (circle) and fibrinoid necrosis (PAS, ×200). (**B**) the glomerulus showed fibrocellular crescent (circle) and endocapillary proliferation with leucocytic infiltrate that surrounded by lymphocytes. Arteriole showed fibrin thrombus (arrow) (H&E, ×200). (**C**) the glomerulus showed fibrous crescent (asterisk) with shrunken tuft (circle) and surrounded by mild lymphocytic infiltrate (arrow) (H&E, ×200). (**D**) arteriole showed endothelial swelling, endothelialitis (arrow) and fibrinoid necrosis (asterisk) (H&E, ×400). (**E**) the interstitium showed moderate lymphocytic infiltrate (arrow) (H&E, ×400). (**F**) the interstitium showed marked IFTA (masson trichrome, ×400). (**G**) the glomerular basement membranes and mesangium showed marked diffuse deposits of IgG (asterisk) and no deposits in the crescent (arrow) (IHC, ×400). (**H**) the glomerular basement membranes and mesangium showed moderate diffuse deposits of C3 (asterisk) and no deposits in the crescent (arrow) (IHC, ×200)

Regarding the renal and extra renal manifestations of the studied patients, oedema was significantly increased in type II compared to type I (*P* = 0.01). Oliguria was discovered to be significantly increased in type II compared to type III (*P* = 0.04). Both type III and type II RPGN groups had significant increases in microscopic hematuria compared to type I RPGN group (*P* = 0.006 and *P* = 0.004, respectively). Type I RPGN had a significant increase in gross hematuria in comparison to type II and type III RPGN groups (*P* = 0.004 and *P* = 0.006, respectively). Fever was significantly found in type II when compared to type I (*P* = 0.02) and type III (*P* < 0.001). Skin rash was significantly increased in type II RPGN compared to type III (*P* < 0.001) and type I RPGN (*P* < 0.001). Hemoptysis was considerably increased in type I RPGN compared to type II RPGN and type III RPGN (*P* < 0.001 and *P* = 0.001, respectively). There was a considerable increase of arthritis in type II RPGN compared to type I and type III RPGN groups (*P* = 0.001 and *P* = 0.02, respectively). 14 patients with type I RPGN, 30 patients with type II RPGN and 11 patients with type III RPGN needed a hemodialysis session at the time of presentation. Both type II and type III RPGN groups had a significant lower level of serum albumin compared to type I RPGN (*P* < 0.001 for each). Serum albumin was also significantly decreased in type III RPGN when compared to type II RPGN (*P* = 0.04). Proteinuria was statistically increased in type II and type III (*P* < 0.001 for each) compared to type I RPGN (Table 1). Immunological profile and histopathological features of the patients had been discussed in details as shown in (Table 2) and (Fig. 1). Because type II RPGN has diverse histological changes and renal outcomes due to different etiologies, we discussed the demographic, clinical, immunological and histopathological characteristics and outcome of patients with type II RPGN separately in (Table 3).

**Table 2:** Immunological profile and histopathological characteristics of the studied RPGN patients

Variables	Type I RPGN *n* = 20	Type II RPGN *n* = 48	Type III RPGN *n* = 33
Low C3 (%)	2 (10%)	42 (87.5%)	0 (0%)
Low C4 (%)	0 (0%)	34 (70.8%)	0 (0%)
+ve ANA (%)	0 (0%)	34 (70.8%)	0 (0%)
+ve AntidsDNA (%)	0 (0%)	34 (70.8%)	0 (0%)
+ve AntiGBM Ab (%)	16 (80%)	0 (0%)	0 (0%)
+ve P-ANCA (%)	0 (0%)	0 (0%)	13 (39.4%)
+ve C-ANCA (%)	0 (0%)	0 (0%)	8 (24.2%)
Total number of glomeruli	20 (4) 15–26	20 (6.75) 12–30	17 (8) 12–24
Number of normal glomeruli	3.0 (1) 1–6	4 (3) 1–14	4 (2) 0–10
Number of sclerotic glomeruli	7 (6) 0–9	4 (4) 0–8	3 (6) 0–7
Number of crescentic glomeruli	11.5 (4) 9–15	11 (3) 7–19	12 (5.5) 5–15
Number of fibrous crescents	3 (2) 1–7	2.5 (1.75) 0–5	2 (3) 0–6
Number of cellular crescents	8.5 (3) 5–12	9 (4.75) 5–16	8 (6) 4–14
Glomerular lesions (%)			
• Endocapillary proliferation (%) • Mesangial proliferation (%) • Neutrophilic infiltration (%) • Glomerular thrombosis (%)	0 (0%) 10 (50%) 12 (60%) 2 (10%)	48 (100%) 26 (54.2%) 32 (66.7%) 0 (0%)	2 (6.1%) 15 (45.5%) 23 (69.7%) 7 (21.2%)
Moderate to severe IFTA (%)	14 (70%)	24 (50%)	16 (48.5%)
TLO formation (%)	10 (50%)	22 (45.8%)	16 (48.5%)
Vascular lesions			
• Fibrinoid necrosis (%) • Degree of severe arteriosclerosis (%)	3 (15%) 8 (40%)	15 (31.3%) 26 (54.2%)	13 (39.4%) 14 (42.4%)
Treatment			
Steroid, CYC as induction then steroid, AZA as maintenance	0 (0%)	30 (62.5%)	29 (87.9%)
Steroid, rituximab	0 (0%)	6 (12.5%)	4 (12.1%)
Steroid, CYC, plasmapheresis as induction then steroid, AZA as maintenance	20 (100%)	12 (25%)	0 (0%)

ANA: antinuclear antibody, ANCA: anti-neutrophil cytoplasmic antibody, Anti-GBM: anti-glomerular basement membrane antibody, IFTA: interstitial fibrosis and tubular atrophy, TLO: tertiary lymphoid organ, RPGN: rapidly progressive glomerulonephritis

Data were expressed as number (%), or median (Interquartile range) and minimum-maximum

**Table 3:** Demographic, clinical, immunological and histopathological characteristics and outcome of type II RPGN

Variables	Lupus nephritis *n* = 34	IgA nephropathy *n* = 4	MPGN *n* = 2	Post-infectious GN *n* = 2	Cryoglobulinemic GN *n* = 6
Age (years)	26.9 (4.5)	37 (3.5)	25 (0.0)	45 (0.0)	50 (4.5)
Gender (%)					
Male Female	4 (11.8%) 30 (88.2%)	2 (50%) 2 (50%)	2 (100%) 0 (0.0%)	0 (0.0%) 2 (100%)	6 (100%) 0 (0.0%)
Edema (%)	26 (76.5%)	2 (50%)	2 (100%)	0 (0.0%)	0 (0.0%)
Microscopic hematuria (%)	34 (100%)	0.0 (0.0%)	2 (100%)	2 (100%)	2 (33.3%)
Macroscopic hematuria (%)	0.0 (0.0%)	4 (100%)	0.0 (0.0%)	0.0 (0.0%)	4 (66.7%)
Oliguria (%)	20 (58.8%)	4 (100%)	2 (100%)	0.0 (0.0%)	4 (66.7%)
Fever (%)	16 (47.1%)	0.0 (0.0%)	0.0 (0.0%)	2 (100%)	0.0 (0.0%)
Skin rash (%)	22 (64.7%)	0.0 (0.0%)	0.0 (0.0%)	2 (100%)	6 (100%)
Hemoptysis (%)	8 (23.5%)	0.0 (0.0%)	0.0 (0.0%)	0.0 (0.0%)	0.0 (0.0%)
Arthritis (%)	26 (76.5%)	0.0 (0.0%)	0.0 (0.0%)	0.0 (0.0%)	4 (66.7%)
Need for HD at presentation (%)	20 (58.8%)	2 (50%)	2 (100%)	2 (100%)	4 (66.7%)
Serum creatinine at presentation (mg/dL)	4.4 (2.1)	4.6 (1.6)	3.5 (0.0)	2.7 (0.0)	5.3 (2.1)
eGFR at presentation (ml/min/1.73^2^)	19.5 (12.8)	15.5 (8.7)	23 (0.0)	20 (0.0)	16.3 (10.7)
Hb (gm/dL)	8.5 (0.9)	9.2 (0.9)	10 (0.0)	9.6 (0.0)	8.8 (0.2)
Serum albumin (gm/dL)	2.2 (0.5)	2.6 (0.6)	2.1 (0.0)	3.1 (0.0)	2.7 (0.4)
Proteinuria (gm/day)	5.8 (2)	4 (0.6)	8 (0.0)	4.7 (0.0)	4.3 (0.2)
Low C3 (%)	34 (100%)	0.0 (0.0%)	0.0 (0.0%)	2 (100%)	6 (100%)
Low C4 (%)	34 (100%)	0.0 (0.0%)	0.0 (0.0%)	0.0 (0.0%)	0.0 (0.0%)
+ve ANA (%)	34 (100%)	0.0 (0.0%)	0.0 (0.0%)	0.0 (0.0%)	0.0 (0.0%)
+ve AntidsDNA (%)	34 (100%)	0.0 (0.0%)	0.0 (0.0%)	0.0 (0.0%)	0.0 (0.0%)
+ve AntiGBM Ab (%)	0.0 (0.0%)	0.0 (0.0%)	0.0 (0.0%)	0.0 (0.0%)	0.0 (0.0%)
+ve P-ANCA (%)	0.0 (0.0%)	0.0 (0.0%)	0.0 (0.0%)	0.0 (0.0%)	0.0 (0.0%)
+ve C-ANCA (%)	0.0 (0.0%)	0.0 (0.0%)	0.0 (0.0%)	0.0 (0.0%)	0.0 (0.0%)
Number of sclerotic glomeruli	3.9 (2.5)	3 (2.3)	2 (0.0)	2 (0.0)	5.3 (1.4)
Number of crescentic glomeruli	11.9 (2.5)	13.5 (6.3)	10 (0.0)	15 (0.0)	11 (1.5)
Number of fibrous crescents	2.5 (1.5)	2.5 (0.6)	1 (0.0)	3 (0.0)	2.7 (0.5)
Number of cellular crescents	9.3 (2.3)	10.5 (6.3)	9 (0.0)	12 (0.0)	8.3 (1.4)
Glomerular lesions (%)					
Endocapillary proliferation (%) Mesangial proliferation (%) Neutrophilic infiltration (%) Glomerular thrombosis (%)	34 (100%) 16 (47.1%) 22 (64.7%) 0.0 (0.0%)	4 (100%) 4 (100%) 4 (100%) 0.0 (0.0%)	2 (100%) 0.0 (0.0%) 0.0 (0.0%) 0.0 (0.0%)	2 (100%) 2 (100%) 2 (100%) 0.0 (0.0%)	6 (100%) 4 (66.7%) 4 (66.7%) 0.0 (0.0%)
Moderate to severe IFTA (%)	18 (52.9%)	2 (50%)	0.0 (0.0%)	2 (100%)	2 (33.3%)
TLO formation (%)	14 (41.2%)	2 (50%)	0.0 (0.0%)	0.0 (0.0%)	6 (100%)
Vascular lesions					
Fibrinoid necrosis (%) Severe arteriosclerosis (%)	11 (32.4%) 18 (52.9%)	0.0 (0.0%) 2 (50%)	0.0 (0.0%) 0.0 (0.0%)	0.0 (0.0%) 0.0 (0.0%)	4 (66.7%) 6 (100%)
Primary outcomes					
ESRD	22 (64.7%)	2 (50%)	0.0 (0.0%)	2 (100%)	4 (66.7%)
Death	10 (29.4%)	0.0 (0.0%)	0.0 (0.0%)	0.0 (0.0%)	2 (33.3%)
Partial remission	8 (23.5%)	0.0 (0.0%)	2 (100%)	0.0 (0.0%)	2 (33.3%)
Complete remission	4 (11.8%)	2 (50%)	0.0 (0.0%)	0.0 (0.0%)	0.0 (0.0%)
Secondary outcomes					
Infections	16 (47.1%)	0.0 (0.0%)	0.0 (0.0%)	2 (100%)	4 (66.7%)
Heart failure	12 (35.3%)	2 (50%)	0.0 (0.0%)	0.0 (0.0%)	0.0 (0.0%)
Respiratory failure	14 (41.2%)	0.0 (0.0%)	0.0 (0.0%)	0.0 (0.0%)	0.0 (0.0%)
Diabetes mellitus	8 (23.5%)	0.0 (0.0%)	0.0 (0.0%)	0.0 (0.0%)	2 (33.3%)

eGFR: estimated glomerular filtration rate, Hb: hemoglobin, HD: hemodialysis, ANA: antinuclear antibody, ANCA: anti-neutrophil cytoplasmic antibody, Anti-GBM: anti-glomerular basement membrane antibody, IFTA: interstitial fibrosis and tubular atrophy, TLO: tertiary lymphoid organ, ESRD: end stage renal disease, Membranoproliferative glomerulonephritis (MPGN)

Data were expressed as number (%) or mean (standard deviation)

### Outcomes in the studied RPGN groups

ESRD was the most frequent primary outcome in our research. Among 101 patients, 60 patients (59.4%) reached ESRD and 32 patients (31.7%) died during follow up. Remission was experienced by 41 patients (40.6%), of whom 11 experienced complete remission and 30 experienced partial remission. In our analysis, the most frequent secondary outcomes included infections, heart failure (HF), respiratory failure (RF) and DM. Infection was the most common secondary outcome affecting 46 patients (45.5%). The main type of infections was pneumonia that led to RF. Cardiac failure affected 40 patients (39.6%), whereas RF occurred in 29 patients (28.7%). DM was identified in 23 patients (22.8%) (Table 4).

**Table 4:** Primary and secondary outcomes in the studied RPGN patients

Variables	Total patients *n* = 101	Type I RPGN *n* = 20	Type II RPGN *n* = 48	Type III RPGN *n* = 33
Primary outcomes				
ESRD	60 (59.4%)	12 (60%)	30 (62.5%)	18 (54.5%)
Death	32 (31.7%)	8 (40%)	12 (25%)	12 (36.4%)
Partial remission	30 (29.7%)	8 (40%)	12 (25%)	10 (30.3%)
Complete remission	11 (10.9%)	0 (0%)	6 (12.5%)	5 (15.2%)
Secondary outcomes				
Infections	46 (45.5%)	10 (50%)	22 (45.8%)	14 (42.4%)
Heart failure	40 (39.6%)	10 (50%)	14 (29.2%)	16 (48.5%)
Respiratory failure	29 (28.7%)	6 (30%)	14 (29.2%)	9 (27.3%)
Diabetes mellitus	23 (22.8%)	6 (30%)	10 (20.8%)	7 (21.2%)

ESRD: end stage renal disease, RPGN: rapidly progressive glomerulonephritis

Data were expressed as number and percentage

### Correlation of mortality and ESRD outcomes with clinical, demographic, and histopathological characteristics

ESRD was positively linked with HTN (*r* = 0.44, *P* < 0.001), oedema (*r* = 0.3, *P* = 0.003), hemoptysis (*r* = 0.36, *P* < 0.001), oliguria (*r* = 0.58, *P* < 0.001) and need for HD at presentation (*r* = 0.66, *P* < 0.001). ESRD was inversely correlated with serum albumin (*P* = 0.001) and Hb level (*P* < 0.001) but favourably correlated with proteinuria (*P* < 0.001). Additionally, there was a positive correlation between ESRD and number of sclerotic glomeruli, number of fibrous crescents, IFTA, arteriosclerosis, TLO formation and mesangial proliferation (*P* < 0.001 for each). Furthermore, ESRD was positively associated with fibrinoid necrosis (*P* = 0.004). Concerning correlation of ESRD with the clinical outcomes, ESRD was positively linked with infections, respiratory failure, HF and DM (*P* < 0.001 for each). There was a positive link between mortality and hemoptysis (*r* = 0.58, *P* < 0.001), oliguria (*r* = 0.41, *P* < 0.001) and need for HD at presentation (*r* = 0.32, *P* = 0.001). Furthermore, mortality was negatively associated with Hb (*r* = − 0.64, *P* < 0.001) and serum albumin (*r* = − 0.34, *P* < 0.001). We found a positive correlation between all-cause mortality and IFTA, number of sclerotic glomeruli, arteriosclerosis, number of fibrous crescents and TLO formation (*P* < 0.001 for each). Fibrinoid necrosis was also positively correlated with the all-cause mortality (*P* = 0.016). Infections, HF, RF, and DM were all positively linked with the mortality outcome (*P* < 0.001 for each) (Table 5).

**Table 5:** Correlation of ESRD and mortality outcomes with demographic, clinical and histopathological characteristics

Variables	ESRD		All-cause mortality	
	r	*P* value	r	*P* value
Age	-0.1	0.27	0.008	0.93
Gender	-0.06	0.53	0.1	0.3
HTN	0.43	**< 0.001**	0.23	**0.018**
Edema	0.3	**0.003**	0.28	**0.004**
Hemoptysis	0.36	**< 0.001**	0.58	**< 0.001**
Oliguria	0.58	**< 0.001**	0.41	**< 0.001**
Creatinine at presentation	0.58	**< 0.001**	0.33	**0.001**
GFR at presentation	-0.6	**< 0.001**	-0.29	**0.003**
Need for HD at presentation	0.66	**< 0.001**	0.32	**0.001**
Hb	-0.66	**< 0.001**	-0.64	**< 0.001**
Albumin	-0.31	**0.001**	-0.34	**< 0.001**
Proteinuria	0.34	**< 0.001**	0.17	0.07
Number of sclerotic glomeruli	0.7	**< 0.001**	0.5	**< 0.001**
Number of crescentic glomeruli	-0.17	0.07	-0.09	0.37
Number of fibrous crescents	0.63	**< 0.001**	0.5	**< 0.001**
Number of cellular crescents	-0.49	**< 0.001**	-0.37	**< 0.001**
IFTA	0.64	**< 0.001**	0.38	**< 0.001**
Arteriosclerosis	0.71	**< 0.001**	0.37	**< 0.001**
Fibrinoid necrosis	0.29	**0.004**	0.24	**0.016**
TLO formation	0.38	**< 0.001**	0.37	**< 0.001**
Mesangial proliferation	0.43	**< 0.001**	0.3	**0.003**
Endocapillary proliferation	0.09	0.35	-0.07	0.43
Neutrophilic infiltration	0.26	**0.008**	0.21	**0.03**
Glomerular thrombosis	0.26	**0.009**	0.16	0.1
Infection	0.43	**< 0.001**	0.57	**< 0.001**
Respiratory failure	0.43	**< 0.001**	0.65	**< 0.001**
Heart failure	0.42	**< 0.001**	0.49	**< 0.001**
Diabetes mellitus	0.35	**< 0.001**	0.49	**< 0.001**

ESRD: end stage renal disease, GFR: glomerular filtration rate, HD: hemodialysis, Hb: hemoglobin, IFTA: interstitial fibrosis and tubular atrophy, TLO: tertiary lymphoid organ

### Comparison of demographic, clinical, histopathological parameters and outcomes in ESRD and non-ESRD patients

We further classified the patients into ESRD (60 patients) and non-ESRD (41 patients) groups based on renal survival. ESRD patients, who consisted of 36 females and 24 males, had a median age of 38 years. The median age of non-ESRD patients who made up of 19 males and 22 females, was 38 years. There was a significant increase in oedema (*P* = 0.003), oliguria (*P* < 0.001), hemoptysis (*P* < 0.001) and need for HD at presentation (*P* < 0.001) in ESRD group in comparison to non-ESRD group. Proteinuria and serum creatinine levels at presentation were both noticeably higher in ESRD patients than in non-ESRD patients. Hb level and serum albumin were considerably lower in the ESRD group (*P* < 0.001 for each). When compared to non-ESRD patients, ESRD patients had a significantly higher number of sclerotic glomeruli and fibrous crescents (*P* < 0.001 for each) and fewer cellular crescents (*P* < 0.001). Mesangial proliferation, moderate to severe IFTA and TLO formation were all significantly increased in ESRD (*P* < 0.001 for each). ESRD patients had more fibrinoid necrosis than non-ESRD patients (*P* = 0.004). Comparing the ESRD group to the non-ESRD group, there was a statistically significant increase in infections, mortality, RF, HF, and DM (*P* < 0.001 for each) (Table 6).

**Table 6:** Demographic, clinical, immunological and histopathological characteristics and clinical outcomes of ESRD and non-ESRD groups

Variables	ESRD group *n* = 60	Non ESRD group *n* = 41	*P* value
Age (years)			
Median (range)	38 (24) 22–65	38 (32) 20–68	0.27
Gender (%)			
Male Female	24 (40%) 36 (60%)	19 (46.3%) 22 (53.7%)	0.52
BMI (Kg/m^2^)	25.8 (1.5)	26.56 (3.7)	
	23–31.64	22–29	0.22
HTN (%)	51 (85%)	18 (43.9%)	**< 0.001**
Edema (%)	40 (66.7%)	15 (36.6%)	**0.003**
Microscopic hematuria (%)	47 (78.3%)	31 (75.6%)	0.75
Macroscopic hematuria (%)	13 (21.7%)	10 (24.4%)	0.75
Oliguria (%)	47 (78.3%)	8 (19.5%)	**< 0.001**
Fever (%)	16 (26.7%)	6 (14.6%)	0.15
Skin rash (%)	24 (40%)	10 (24.4%)	0.1
Hemoptysis (%)	26 (43.3%)	4 (9.8%)	**< 0.001**
Arthritis (%)	30 (50%)	16 (39%)	0.27
Need for HD at presentation (%)	49 (81.7%)	6 (14.6%)	**< 0.001**
Serum creatinine at presentation (mg/dL)	5.4 (2.4)	2.7 (1.0)	
	1.2–8	1.2–5.8	**< 0.001**
GFR at presentation (ml/min/1.73^2^)	11 (5)	27 (6.5)	
	6–81	9–63	**< 0.001**
Hb (gm/dL)	8.0 (1.3)	9.5 (1.1)	
	6.6–9.6	8–10.5	**< 0.001**
Serum albumin (gm/dL)	2.0 (0.9)	2.7 (0.9)	
	1.6–3.1	1.8–3.1	**0.002**
Proteinuria (gm/day)	5 (2.1)	4.0 (1.3)	
	2.5–12	2–8	**0.001**
Low C3 (%)	30 (50%)	14 (34.1%)	0.11
Low C4 (%)	22 (36.7)	12 (29.3%)	0.44
+ve ANA (%)	22 (36.7)	12 (29.3%)	0.44
+ve AntidsDNA (%)	22 (36.7)	12 (29.3%)	0.44
+ve AntiGBM (%)	12 (20%)	4 (9.8%)	0.16
+ve P-ANCA(%)	7 (11.7%)	6 (14.6%)	0.66
+ve C-ANCA (%)	6 (10.%)	2 (4.9%)	0.35
Total number of glomeruli	20 (6)	17 (5.5)	
	12–26	12–30	**0.004**
Number of normal glomeruli	3 (2)	4 (2.5)	
	0–11	2–14	**0.028**
Number of sclerotic glomeruli	6 (3)	1.0 (2)	
	1–9	0–7	**< 0.001**
Number of crescentic glomeruli	11(3)	13.0 (4)	0.07
	7–16	5–19	
Number of fibrous crescents	3.0 (1)	1.0 (1)	
	1–7	0–3	**< 0.001**
Number of cellular crescents	8.0 (2.75)	11.0 (4.0)	
	4–12	5–16	**< 0.001**
Glomerular lesions (%)			
Endocapillary proliferation (%) Mesangial proliferation (%) Glomerular thrombosis (%) Neutrophilic infiltration (%)	32 (53.3%) 41 (68.3%) 9 (15%) 46 (76.7%)	18 (43.9%) 10 (24.4%) 0 (0%) 21 (51.2%)	0.35 **< 0.001** **0.009** **0.008**
Moderate to severe IFTA (%)	48 (80%)	6 (14.6%)	**< 0.001**
TLO formation (%)	38 (63.3%)	10 (24.4%)	**< 0.001**
Vascular lesions			
Fibrinoid necrosis Degree of severe arteriosclerosis (%)	25 (41.7%) 46 (76.7%)	6 (14.6%) 2 (4.9%)	**0.004** **< 0.001**
Infections (%)	38 (63.3%)	8 (19.5%)	**< 0.001**
Heart failure (%)	34 (56.7%)	6 (14.6%)	**< 0.001**
Respiratory failure (%)	27 (45%)	2 (4.9%)	**< 0.001**
Diabetes mellitus (%)	21 (35%)	2 (4.9%)	**< 0.001**

ANA: antinuclear antibody, ANCA: anti-neutrophil cytoplasmic antibody, Anti-GBM: anti-glomerular basement membrane antibody, BMI: body mass index, ESRD: end stage renal disease, GFR: glomerular filtration rate, HD: hemodialysis, Hb: hemoglobin, IFTA: interstitial fibrosis and tubular atrophy, SD: standard deviation, TLO: tertiary lymphoid organ. Data were expressed as number (%), mean ± standard deviation or median and range

### Predictors of ESRD and all-cause mortality in the patients

**Fig. 2:**
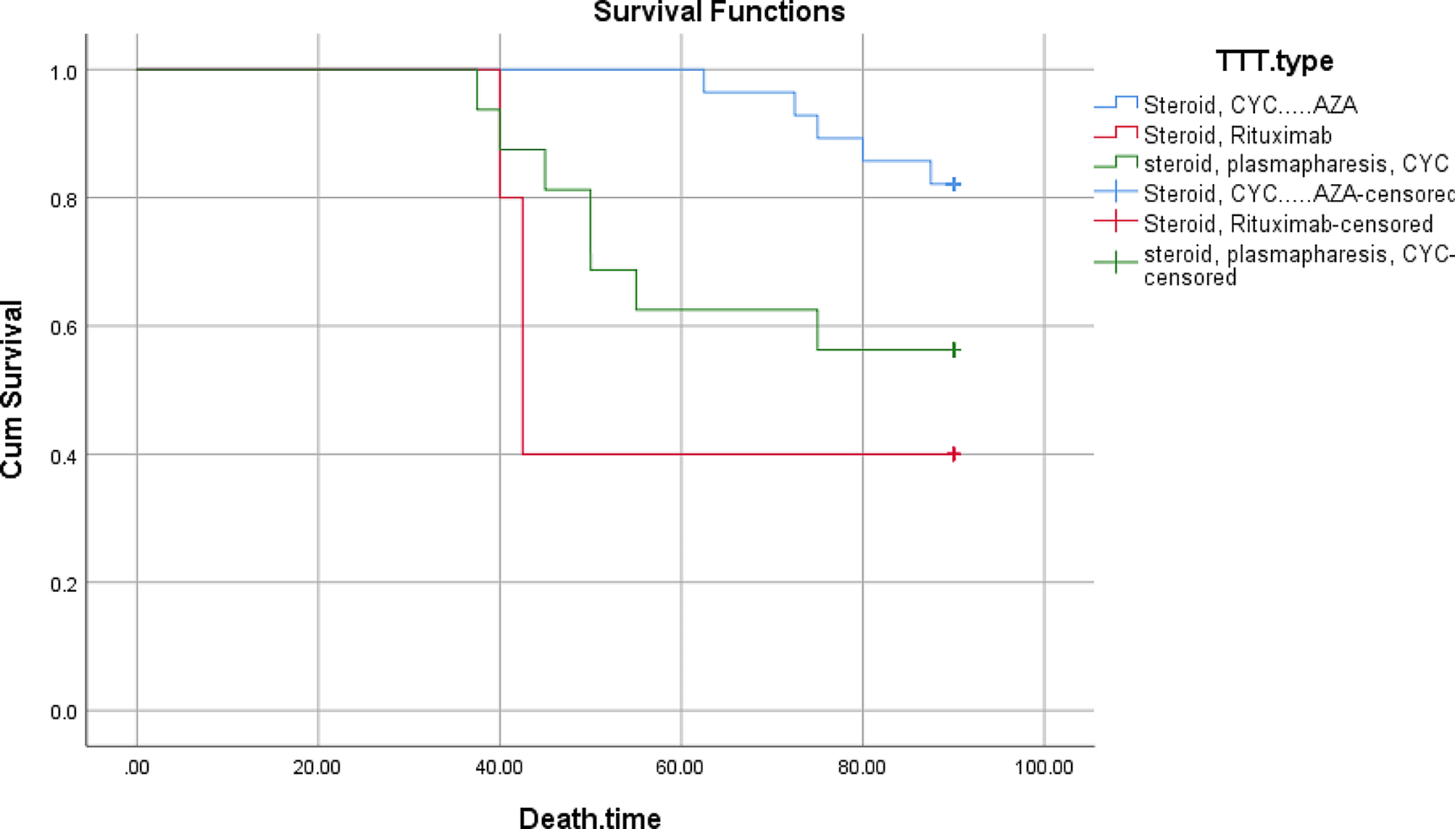
**Comparison of the patient survival by treatment regimens**: Kaplan-Meier survival analysis for a 90-month mortality showing that patients treated with steroid, cyclophosphamide and azathioprine had the best patient survival, whereas those treated with steroids and rituximab had the worst prognosis for patient survival (log-rank *P* < 0.001)

**Fig. 3:**
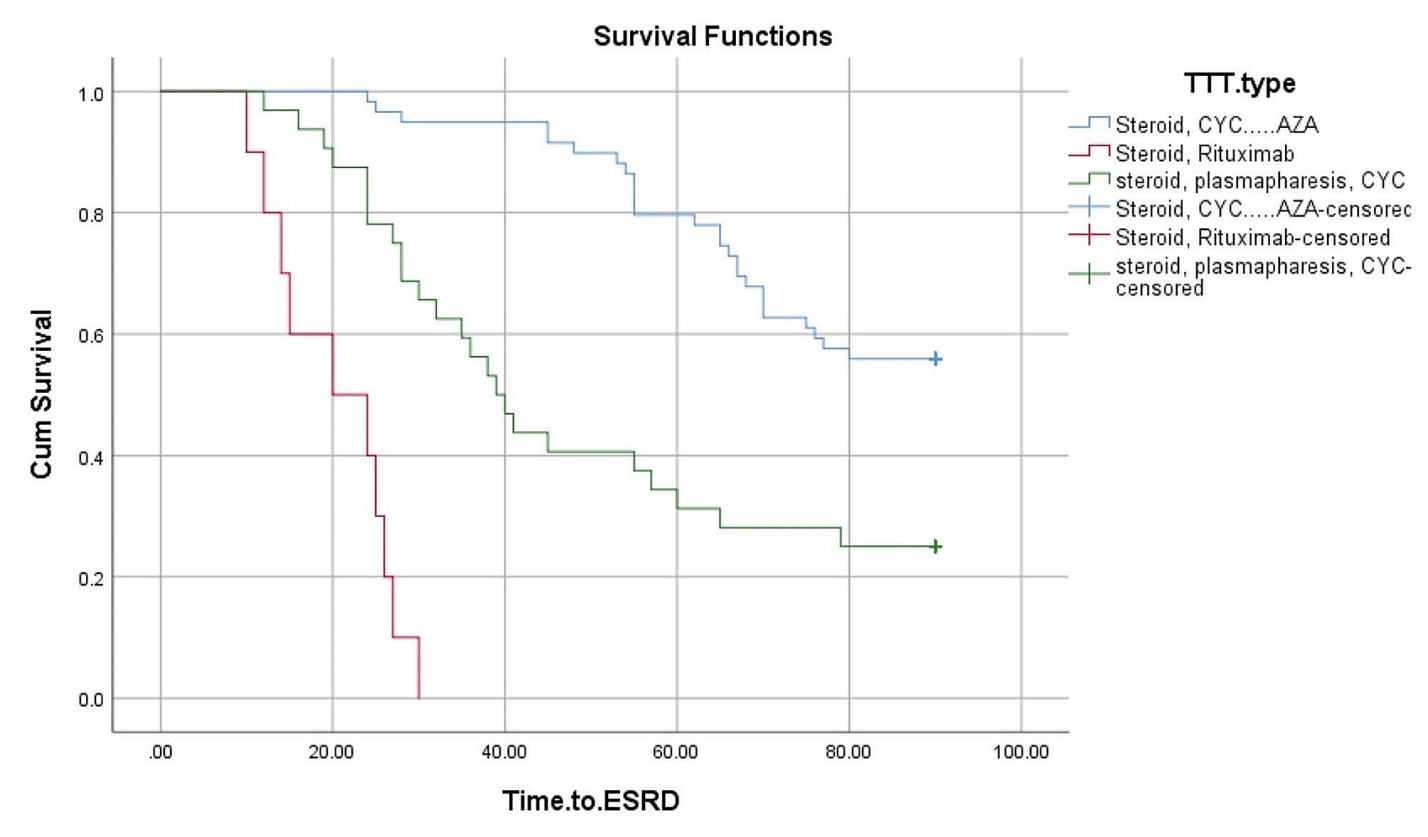
**Comparison of the renal survival by treatment regimens**: Kaplan-Meier survival analysis showed that patients treated with steroid, cyclophosphamide and azathioprine had the best prognosis for renal survival, whereas those receiving steroids and rituximab had the worst renal survival (log-rank *P* < 0.001)

The significant predictors of renal loss on logistic univariate regression were oliguria (*P* < 0.001), proteinuria (*P* = 0.002), eGFR at presentation (*P* = 0.002), need for hemodialysis at presentation (*P* < 0.001), number of sclerotic glomeruli (*P* < 0.001), number of fibrous crescents (*P* < 0.001), TLO formation (*P* < 0.001) and moderate to severe IFTA (*P* < 0.001). Logistic multivariate regression revealed that number of sclerotic glomeruli (*P* = 0.036) and IFTA (*P* = 0.008) were reliable predictors of ESRD (Table 7). By using univariate Cox regression, it was found that death was independently associated with hemoptysis, RF, HF, TLO development, IFTA, requirement for HD at presentation, percentage of sclerotic glomeruli, and fibrous crescents. Multivariate Cox regression showed that respiratory failure (HR: 0.03, *P* < 0.001), heart failure (HR: 0.20, *P* = 0.004), infections (HR: 0.17, *P* = 0.02) and eGFR (HR: 0.97, *P* = 0.02) were reliable risk factors associated with mortality in our study (Table 8). 59 patients were treated with steroid and cyclophosphamide to induce remission and oral azathioprine and steroid to maintain remission. Of them 12 died. Rituximab was given to 10 patients and 6 of them died. 32 patients received plasmapheresis, steroid and cyclophosphamide as an induction therapy followed by steroid and oral azathioprine as a maintenance therapy. Of them fourteen died. A Kaplan-Meier survival analysis showed that patients treated with induction therapy with steroid and cyclophosphamide, followed by maintenance therapy with steroid and azathioprine had the best prognosis for patient and renal survival, while those receiving steroids and rituximab had the worst prognosis for both renal and patient survival (log-rank *P* < 0.001, for each) (Figs. 2 and 3).

**Table 7:** Logistic regression to assess predictors of ESRD in the studied patients

Variables	Univariate logistic regression				Multivariate logistic regression			
	*P* value	Exp (B)	95% CI		*P* value	Exp (B)	95% CI	
			Lower	Upper			Lower	Upper
eGFR at biopsy	**0.002**	0.94	0.90	0.98	0.09	0.88	0.75	1.02
Proteinuria	**0.002**	1.71	1.22	2.39	0.11	2.86	0.78	10.52
Fibrous crescents	**< 0.001**	4.11	2.35	7.16	0.59	1.52	0.33	6.92
IFTA (moderate-severe)	**< 0.001**	0.04	0.02	0.12	**0.008**	0.002	0.00	0.21
TLO formation	**< 0.001**	0.19	0.08	0.00	0.17	0.13	0.01	2.39
Oliguria	**< 0.001**	0.07	0.02	0.18	0.49	3.25	0.11	92.65
Need for HD	**< 0.001**	0.04	0.01	0.11	0.37	0.28	0.02	4.48
Sclerotic glomeruli	**< 0.001**	2.39	1.76	3.25	**0.036**	2.06	1.05	4.06

HD: hemodialysis, IFTA: interstitial fibrosis and tubular atrophy, TLO: tertiary lymphoid organ, eGFR: estimated glomerular filtration rate, CI: confidence interval

**Table 8:** Cox regression study to determine predictors of mortality in the patients

Variables	Univariate Cox regression				Multivariate Cox regression			
	*P* value	HR	95% CI		*P* value	HR	95% CI	
			Lower	Upper			Lower	Upper
Hemoptysis	**< 0.001**	0.11	0.05	0.23	0.13	0.37	0.10	1.32
Infections	**< 0.001**	0.08	0.03	0.23	**0.02**	0.17	0.04	0.79
Respiratory failure	**< 0.001**	0.08	0.04	0.17	**< 0.001**	0.03	0.01	0.15
Heart failure	**< 0.001**	0.17	0.07	0.37	**0.004**	0.20	0.07	0.59
IFTA (moderate - severe)	**< 0.001**	0.2	0.08	0.49	0.76	1.27	0.27	5.99
Fibrous crescents	**< 0.001**	1.83	1.49	2.25	0.94	0.99	0.65	1.48
TLO formation	**0.001**	0.27	0.12	0.59	0.6	1.28	0.51	3.21
Sclerotic glomeruli	**< 0.001**	1.43	1.24	1.66	0.45	1.14	0.81	1.62
Need for HD	**0.002**	0.27	0.11	0.62	0.29	2.23	0.50	9.90
eGFR at biopsy	0.14	0.98	0.95	1.01	**0.02**	0.97	0.945	1.00

HR: hazard ratio, HD: hemodialysis, IFTA: interstitial fibrosis and tubular atrophy, TLO: tertiary lymphoid organ, eGFR: estimated glomerular filtration rate, CI: confidence interval

## Discussion

3340 renal biopsies were conducted in our centre over a period of 7.5 years, and 101 (3%) of those were found to have RPGN. This is similar to previously reported studies from India [3, 6, 19]. Our study discovered that type II was the most common cause of RPGN, followed by type III and type I. Different studies from Saudi Arabia, China, and an Indian study have shown that type II crescentic GN was the most common [7, 19, 20]. Similar results were shown by Alexander et al. who found that type II was the most prevalent (46.5%) followed by type III and type I [3]. Lupus nephritis (33.7%) was the most frequent cause of type II RPGN followed by cryoglobulinemic GN (5.9%), IgA nephropathy (4%), post-infectious GN (2%) and MPGN (2%). This is comparable to a large Chinese review in which lupus nephritis (34%) was recognised as the most common etiology of crescentic GN [6]. Infection rates and the frequency of SLE have been linked to the higher incidence of type II RPGN in these regions. Contradictory studies in different parts of the world have shown that type III was the most common RPGN [21, 22].

In comparison to type II and type I RPGN, type III RPGN had a noticeably greater average age. This finding is similar to Alexander et al. who discovered that patients with type III RPGN were older than those with type I and type II RPGN (*P* = 0.03 and *P* = 0.003, respectively). Gender differences in the majority of type II RPGN have been shown to be female-predominant. This is comparable to the study by Alexander et al. [3]. In several researches, the distribution of gender in type I and type III crescentic GN had varied [7, 10, 19, 20, 22]. Oliguria was discovered to be significantly increased in type II compared to type III. This contradicts Parry et al.’s study that found an insignificant difference in oliguria between the all groups [23]. Our patients in type III and type II RPGN groups had significantly higher rates of microscopic or gross hematuria. This goes against the findings of Parry et al. and Alexander et al. who claimed that there was no variation in hematuria among the RPGN groups [3, 23]. Requirement for hemodialysis was significantly higher in type I and type II groups compared to type III. This resembles the findings of Parry et al. who claimed that type I patients needed more hemodialysis sessions than type II patients (*P* = 0.035) [23]. This is also in agreement with Eksin et al. who claimed that type II required more hemodialysis sessions than type III (*P* = 0.01) [24].

Immune complex mediated RPGN had a significantly greater rate of skin rash than type I and type III. This agrees with the studies of Parry et al. and Alexander et al. that reported that skin rash was found significantly in type II compared to type III and type I [3, 23]. Anti-GBM illness had significantly more hemoptysis than type II or type III RPGN. This contradicts Alexander et al.’s findings, which showed that hemoptysis was more common in type III than in type II and type I [3]. Our study found that arthritis was more common in type II RPGN affecting 15 patients (62.5%). This is contrary to the findings of Alexander et al. who didn’t discover significant differences in arthritis between the groups [3].

All RPGN groups had reduced average Hb levels, but there was no discernible difference in Hb levels between the groups. This is comparable to earlier researches [23, 24]. Anemia occurs in variable degrees in patients with renal disorders [25]. This was also seen in the patients of our current study. This is in disagreement with Wu et al. who discovered that type II patients had less severe anemia than those who had type I or type III [10]. Our study reported that serum albumin levels in type II and type III groups were significantly lower than in type I group. Comparing type III to type II, serum albumin levels were also noticeably lower in type III. Proteinuria levels in type II and type III were greater than in type I. This is consistent with the findings of the Parry et al. study, which showed that type II crescentic GN had the lowest mean serum albumin (*P* = 0.006) and the highest mean proteinuria (*P* < 0.001) [23]. This is in agreement with the research by Wu et al. who found that type III patients had less proteinuria [10]. This also goes against the findings of Wu et al. who discovered no appreciable variations in albumin levels between the groups [10].

We didn’t discover any significant variations in the proportions of glomeruli exhibiting fibrous crescents and cellular crescents between all RPGN groups in our current study, which was in line with the results of previous researches [7, 9, 10, 26]. Parry et al. discovered contradicting data, reporting that crescents were present in 75.1 ± 18.3 of glomeruli, with the largest percentage occurring in type I RPGN (87 ± 15.2, *P* = 0.04). Additionally, Parry et al. demonstrated that in all three groups of RPGN, fibrocellular crescents were the most prevalent type of crescent [23]. Endocapillary proliferation was not seen in anti-GBM illness, but it was found in all patients with type II RPGN and two patients with type III RPGN. In 54.2% of patients with immune complex-mediated RPGN and 50% of patients with anti-GBM illness, mesangial proliferation was found. Additionally, 45.5% of patients with pauci-immune RPGN had it. Similar data were discovered by Parry et al. who revealed that the majority of type II RPGN showed endocapillary proliferation (97.2%, *P* < 0.001) and mesangial proliferation (69.4%, *P* < 0.001) [23]. Similar findings were also reported by studies of Alexander et al. and Ganesan et al. [3, 27]. In our research, neutrophil infiltration was present in 66.7%, 69.7%, and 60% of patients with type II, type III, and type I RPGN, respectively. This is in line with the findings of Alexander et al. who demonstrated that type II RPGN had considerably higher levels of neutrophilic infiltrates (50.5%, *P* = 0.01) [3]. This disagrees with the study of Parry et al. that found that type I RPGN (81.2%, *P* = 0.008) had the largest proportion of glomeruli with neutrophilic infiltrates, followed by type II (52.7%) and type III (30.3%) [23]. Our data showed that a moderate to severe IFTA was present in 70% of patients with anti-GBM illness. Additionally, it was discovered in 50% of patients with type II RPGN and 48.5% of type III RPGN patients. This supports the findings of Ganesan et al. research, which showed that non-immune complex mediated crescentic GN had a significantly greater rate of moderate to severe IFTA than immune complex mediated crescentic GN (*P* = 0.009) [27]. Our study explained that TLO formation was found in 50%, 45.8% and 48.5% of patients with type I, type II and type III RPGN, respectively.

We found that glomerular thrombosis was discovered in 2 patients with type I RPGN (10%) and 7 patients with type III RPGN (21.1%). It wasn’t reported in patients with type II RPGN. Alexander et al. reported glomerular thrombosis in 3 patients with type II RPGN. Alexander et al. also found that patients with type I and type III RPGN didn’t exhibit glomerular thrombosis [3]. There is an interaction between the complement system, platelets, the coagulation system and neutrophils. (i) Coagulation factors, including fibrinogen, factor XIII, and prothrombin, can be cleaved by MASPs. (ii) C5a and C5b-9 enhance blood thrombogenicity through upregulation of tissue factor in neutrophils and endothelial cells. (iii) C5a stimulates secretion of ultra large Von Willebrand factor and expression of P-selectin. Based on these findings, it appears that C5a plays a significant role as an inflammatory mediator between neutrophils and glomerular endothelial cells during the acute phase of inflammation [28]. We also reported fibrinoid necrosis in 15%, 31.3% and 39.4% of patients with type I, type II and type III RPGN, respectively. This resembles Gupta et al. who found fibrinoid necrosis in 54.5%, 23% and 29% of patients with type I, type II and type III RPGN, respectively [9]. This contradicts Nagaraju et al. who discovered that RPGN patients did not exhibit arterial fibrinoid necrosis [6]. Complement activation is responsible for the active lesions that include intravascular neutrophil karyorrhexis or NETosis, immunothrombosis and fibrin deposition, endothelial and mesangial cell proliferation, glomerular leukocytic infiltrates, vascular fibrinoid necrosis, and cellular crescents. 50–80% of cases involve the ANCA. The ANCA is directed against myeloperoxidase (MPO), proteinase 3 (PR3), or both. While the exact process underlying the development of ANCA remains unclear, it is known that autoantibodies cause neutrophils to become activated, thereby damaging the glomerular capillary wall. An alternate pathway is primarily responsible for the activation of both local and systemic complement. Additionally, the cytokines that includes TNF-α, play a significant role in the pathogenesis. Production of anti-plasminogen and plasminogen activator autoantibodies can inhibit fibrinolysis and predispose to fibrinoid necrosis and thrombophilia [29].

In our study, the primary outcome with the highest frequency was ESRD. Out of 101 patients, 60 (59.4%) developed ESRD, and 32 (31.7%) died while being followed up. 41 patients (40.6%) went into remission, with 30 going into partial remission and 11 going into complete remission. Nagaraju et al. study reported similar findings. He demonstrated that 8 patients out of 29 died, making up 27.6% of the total mortality. At the end of the observation period, 34.5% of patients had complete or partial remission, and 30% had ESRD necessitating ongoing hemodialysis [6]. It resembles Rampelli et al.’s findings, which showed that 48.6% of patients developed ESRD and 18.9% of patients died [19]. Sharma et al. showed similar results. They reported that 12.5% and 22.5% of patients experienced complete and partial remission, respectively. They demonstrated that 10 patients died during follow up [2]. According to the research by Erdogmus et al., 14 patients (13.5%) died and 35 patients (34%) advanced to ESRD by the conclusion of the 30-month follow-up period [30]. Furthermore, Alsuheili et al. reported similar findings [31]. Regarding the secondary outcomes in our analysis, infection affected 46 patients (45.5%) and was the most frequent secondary result. Pneumonia was the primary infection type that resulted in RF. 40 patients (39.6%) experienced cardiac failure, whereas 29 individuals (28.7%) experienced RF. In 23 patients (22.8%), DM was found. These results are similar to findings of sharma et al. that showed that infections affected more than half of the patients (55%). Diabetes and heart failure were noted in 30% and 25% of patients, respectively. The most prevalent infection among the patients was pneumonia (40.9%), which was followed by septicaemia (27.9%) [2]. Furthermore, infections, heart failure, and diabetes were all present in 67.2%, 29.09%, and 29.09% of the patients, respectively, according to Wangnoo et al. [5]. According to Sandhu’s study, infections and neutropenia (55 and 40%, respectively) were the two most frequent secondary outcomes [32].

On the basis of renal survival, we further divided the patients into ESRD and non-ESRD groups. Furthermore, there was a significant increase in oedema, HTN, oliguria, need for HD at presentation, serum creatinine levels at admission and proteinuria in ESRD group. Serum albumin and Hb level were considerably lower in the ESRD group. ESRD patients had a significantly higher number of sclerotic glomeruli and fibrous crescents and fewer cellular crescents. Mesangial proliferation, moderate to severe IFTA and TLO formation were all significantly increased in ESRD group. Similar results were obtained in the study by Lim et al. He found that HTN (*P* = 0.02), serum creatinine (*P* < 0.001), percentage of normal glomeruli (*P* < 0.001), percentage of sclerotic glomeruli (*P* = 0.002), arteriosclerosis (*P* = 0.002), TLO development (*P* < 0.001), and severe tubular atrophy (*P* < 0.001) were all significantly higher in ESRD patients [33]. In addition, the study by Eksin et al. discovered that ESRD patients reported a considerable increase in the percentage of cellular glomeruli (*P* < 0.001), fibrocellular glomeruli (*P* < 0.001), and the requirement for hemodialysis at admission (*P* = 0.01). Additionally, they found that people with ESRD had much decreased Hb [24].

TLO formation, also known as lymphoid neogenesis, denotes nodular inflammatory cells infiltration in non-lymphoid organs. Histopathologically, TLOs are identified as having aggregated tiny cells, such as T and B lymphocytes, and other cellular elements made up of bigger follicular structures. TLOs have been detected in multiple chronic inflammatory states, as GN, rheumatoid arthritis, membranous nephropathy and lupus nephritis [33]. TLO production is a sign of a persistent inflammatory state and encourages local immunological responses; for these reasons, TLOs play an essential role in progression of the disease [33]. Data showed that the poor renal survival in patients with TLO formation even after controlling for other risk factors; is likely because, TLOs, like other autoimmune illnesses, are produced by tissue antigens released by the destructive inflammatory process associated with RPGN. As a result, TLO development stimulates autoimmune response, which is damaging to renal parenchyma of RPGN patients. Patients with TLO development in particular advanced to ESRD early, necessitating more aggressive treatment for these patients at the time of diagnosis [34].

According to the findings of our study, ESRD is positively correlated with HTN, oedema, hemoptysis, oliguria, the requirement for HD at presentation and proteinuria. The number of sclerotic glomeruli, the number of fibrous crescents, TLO development, IFTA, arteriosclerosis and mesangial proliferation were all positively linked with ESRD. Additionally, serum albumin, and Hb level had an adverse relationship with ESRD. Oliguria, proteinuria, the percent of sclerotic glomeruli, the requirement for hemodialysis at presentation, eGFR at presentation, the development of TLO, and IFTA were significant predictors of renal loss in the univariate logistic regression. The results of multivariate logistic regression analysis showed that IFTA and the quantity of sclerotic glomeruli were accurate indicators of ESRD. Mortality outcome had a positive association with hemoptysis, the requirement for HD at presentation, oliguria, IFTA, sclerotic glomeruli number, arteriosclerosis, fibrous crescents number, TLO development and mesangial proliferation. Additionally, Hb and serum albumin had a negative link with mortality. Multivariate Cox regression demonstrated that eGFR at presentation, respiratory failure, HF, infections were reliable factors linked to mortality in our study.

The study by Nagaraju et al. reported that oliguria, the need for hemodialysis at presentation, and high serum creatinine at admission were the major risk factors linked with death and renal loss on univariate regression analysis. In contrast to our investigation, histopathological characteristics such IFTA or the presence of fibrous/fibrocellular crescents had no influence on the results [6]. Both the need for dialysis at the time of diagnosis (OR: 0.07, *P* = 0.018) and serum creatinine (OR: 6.37, *P* = 0.019) were strong indicators of renal loss, according to univariate regression in Eksin et al. study [24]. According to Erdogmus et al.‘s research, IFTA score of more than 25%, as well as a serum creatinine of 3.5 mg/dl or higher, were risk factors for ESRD. These factors were revealed by Cox regression study for renal survival [30]. In a study by Parry et al., risk factors for a poor renal outcome were examined using univariate regression. Oliguria (HR: 2.9, *P* < 0.001), glomerular sclerosis (HR: 1.8, *P* = 0.01), a larger percentage of crescents (HR: 1.3, *P* = 0.002), and moderate/severe IFTA (HR: 1.7, *P* = 0.025) were linked with worse renal outcomes [23]. ESRD was significantly predicted by low hematocrit levels, higher creatinine levels, tubular necrosis, interstitial fibrosis, abnormal glomeruli percentage, cellular crescents, glomeruli with extracapillary proliferation, and overall glomerulosclerosis, according to a reliable study by Kapitsinou et al. Predictors for death were comparatively similar [35]. Sandhu reported that mortality was positively linked with infections (*P* = 0.05). 10 patients (25%) died during the study’s duration. Infections accounted for 50% of causes of death, followed by cardiovascular disease (30%), stroke (10%), and unknown in 10% of cases [32]. Alexander et al.‘s research revealed that serum creatinine at presentation (*P* < 0.001), oliguria (*P* < 0.001), percent of crescents (*P* < 0.001), mesangial proliferation (*P* = 0.01), and IFTA (*P* < 0.001) were all shown to be ESRD predictors by univariate logistic regression. Percent of crescents (*P* = 0.01), IFTA (*P* < 0.001) and oliguria (*P* = 0.001) were all identified as ESRD predictors by multivariate regression [3].

Our patients treated with cyclophosphamide had the best kidney and patient survival, whereas those receiving rituximab had the worst outcomes. 6 of rituximab treated patients died due to infections. Similar findings were found by Aksoy et al. study in SLE patients treated with rituximab. Akosy et al. discovered that 20 individuals who received rituximab treatment had a known cause of death. The leading cause of mortality (10/20, 50%) was infection, which was followed by ischemic heart disease (5/20, 25%), and cancer (3/20, 15%) [36]. Additionally, serious infections were linked to higher mortality in the six months after the 1st rituximab (HR: 4.97,*P* < 0.001) [37]. This is consistent with Lee et al. study that revealed that the usage of cyclophosphamide was an independent predictor of renal and patient survival by survival analysis [38]. Additionally, the impact of cyclophosphamide therapy on patient survival is consistent with the findings of previous investigations [39, 40]. Our data were consistent with Ishikawa et al. study that found that 8.5% of the rituximab group experienced poor renal outcomes compared with 2.4% of the cyclophosphamide group (*P* < 0.01) [41]. The RITUXIVAS trial (*n* = 44) found contradictory findings. It involved 33 rituximab-treated patients with varying degrees of renal impairment (median eGFR 20 mL/min/1.73 m^2^, 24% dialysis dependent). Adverse events and outcomes were comparable to those of patients receiving traditional glucocorticoid and cyclophosphamide induction therapy [42].

Our study is the first in North Africa to identify these various clinical, histopathological and treatment predictors of RPGN over a prolonged period of follow up. Our study is the first to determine the secondary outcomes of RPGN such as respiratory failure, DM, heart failure and infections in addition to the primary outcomes. Additionally, we also extensively discussed the treatment regimens and their role in prediction of mortality and ESRD outcomes by survival analysis. We are recently discussed TLO and its role in progression of RPGN. We found that it was a strong predictor of ESRD. All of these findings can provide new insights in histopathological and treatment predictors of outcome of RPGN in North Africa especially Egypt where there are little data on RPGN. A retrospective design is a limitation of our study, but we avoided all bias introduced by the retrospective design as selection bias, information bias and confounding.

## Conclusions

This study offers important details regarding the spectrum of RPGN diseases. Over a seven-year period, RPGN was discovered in 3% of native kidney biopsies. The most typical types that caused RPGN were type II, followed by type III, and type I. Type II RPGN was frequently caused by lupus nephritis. Different clinical characteristics can be seen in RPGN patients. ESRD, which affected 59.4% of patients, was the primary outcome and 32 patients (31.7%) died while being followed up. 41 patients (40.6%) experienced remission. Infections, HF, RF, and DM were the most frequent secondary outcomes. Infection was the most common secondary outcome. The main type of infections was pneumonia that led to RF. Proteinuria, eGFR at presentation, the percent of sclerotic glomeruli, and histopathological findings, such as severe IFTA and glomerulosclerosis were significant predictors of renal loss. RF, HF, infections and eGFR at presentation were all significant risk factors for mortality. Thus to improve outcome, an early referral to nephrologist for early diagnosis and treatment is essential for renal and patient survival.

## Data Availability

Availability of Data and materials: The corresponding author can provide the datasets used and/or analyzed during the current research upon reasonable request.

## Abbreviations

ANA: Antinuclear antibody
ANCAs: Antineutrophil cytoplasmic antibodies
Anti-GBM: Anti-glomerular basement membrane
BMI: Body mass index
C3: Complement 3
CI: Confidence interval
DM: Diabetes mellitus
eGFR: estimated glomerular filtration rate
ESRD: End stage renal disease
HD: Hemodialysis
HF: Heart failure
HR: Hazard ratio
IFTA: Interstitial fibrosis with tubular atrophy
IgA: Immunoglobulin A
IgG: Immunoglobulin G
IHC: Immunohistochemistry
IQR: Interquartile ranges
LM: Light microscopy
MPGN: Membranoproliferative glomerulonephritis
RF: Respiratory failure
RPGN: Rapidly progressive glomerulonephritis
TLO: Tertiary lymphoid organ
